# MERS-CoV in Camels but Not Camel Handlers, Sudan, 2015 and 2017

**DOI:** 10.3201/eid2512.190882

**Published:** 2019-12

**Authors:** Elmoubasher Farag, Reina S. Sikkema, Ahmed A. Mohamedani, Erwin de Bruin, Bas B. Oude Munnink, Felicity Chandler, Robert Kohl, Anne van der Linden, Nisreen M.A. Okba, Bart L. Haagmans, Judith M.A. van den Brand, Asia Mohamed Elhaj, Adam D. Abakar, Bakri Y.M. Nour, Ahmed M. Mohamed, Bader Eldeen Alwaseela, Husna Ahmed, Mohd Mohd Alhajri, Marion Koopmans, Chantal Reusken, Samira Hamid Abd Elrahman

**Affiliations:** Ministry of Public Health, Doha, Qatar (E. Farag, M.M. Alhajri);; Erasmus Medical Centre, Rotterdam, the Netherlands (R.S. Sikkema, E. de Bruin, B.B.O. Munnink, F. Chandler, R. Kohl, A. van der Linden, N.M.A. Okba, B.L. Haagmans, M. Koopmans, C. Reusken);; University of Gezira, Wad Medani, Sudan (A.A. Mohamedani, S.H. Abd Elrahman);; Utrecht University, Utrecht, the Netherlands (J.M.A. van den Brand);; Blue Nile National Institute for Communicable Diseases, Wad Medani (A.M. Elhaj, A.D. Abakar, B.Y.M. Nour, A.M. Mohamed, S.H. Abd Elrahman);; Tamboul Camel Research Centre, Tamboul, Sudan (B.E. Alwaseela, H. Ahmed);; National Institute for Public Health and the Environment, Bilthoven, the Netherlands (C. Reusken)

**Keywords:** Middle East respiratory syndrome coronavirus, coronavirus, viruses, zoonoses, serology, seropositive, S1 protein, PCR, veterinary medicine, One Health, public health, *Camelus*, camels, livestock, camel handlers, humans, bats, live animal market, Sudan, Qatar

## Abstract

We tested samples collected from camels, camel workers, and other animals in Sudan and Qatar in 2015 and 2017 for evidence of Middle East respiratory syndrome coronavirus (MERS-CoV) infection. MERS-CoV antibodies were abundant in Sudan camels, but we found no evidence of MERS-CoV infection in camel workers, other livestock, or bats.

Middle East respiratory syndrome coronavirus (MERS-CoV) is a zoonotic virus from camels that can cause serious respiratory disease and death in humans (*1*). Camel populations across the Middle East and Africa are highly seropositive. However, the only known human cases of clinical MERS-CoV infection in Africa were related to travel from Qatar and Saudi Arabia (https://ecdc.europa.eu/sites/portal/files/media/en/publications/Publications/RRA_MERS-CoV_7th_update.pdf), and serologic evidence for infections in humans resulting from camel exposure in Africa is limited (*2*).

The only published report of MERS-CoV circulation in camels in Sudan involved the testing of camel samples from 1983; that study found a seroprevalence of 82% (49/60) (*3*). Two publications from Egypt describe evidence of possible MERS-CoV circulation in Sudan, reporting a seroprevalence of 91% (543/594) in camels originating from Sudan and a seroprevalence of 92% (48/52), combined with a reverse transcription PCR positivity rate of 5.6%, in camels originating from Ethiopia and Sudan (*3*). Neither study presented conclusive evidence for MERS-CoV circulation in Sudan. Here, we provide the results of a study conducted in the Butana region of Al Gezira, Sudan, to investigate the local point prevalence of MERS-CoV and MERS-CoV antibodies among camel handlers, camels, and other animals in 2015 and 2017. We also report the results of a MERS-CoV screening in camels from Sudan sampled in Qatar directly upon importation.

We collected samples from humans and animals at a live animal market, an outdoor slaughter area adjacent to that market, and the Tamboul Camel Research Centre (TCRC), all located in Tamboul, Sudan. Overall, ≈1,660 camels and additional other livestock are usually present at the animal market; these camels come from individual small farms, where they are largely kept under free-roaming conditions. At the TCRC, ≈100 camels are generally present and kept out of contact with other camels. Before their arrival at the TCRC, they were herded on the Butana Plain. We also collected samples from 90 Sudan camels that were imported into Qatar in 2015. After arriving at the Hamad International Airport in Doha, Qatar, these camels were directly transported to the Al Shahaniya animal market in Doha. We sampled them immediately after their arrival. We stored all samples locally (1–1.5 years in Sudan, 1 month in Qatar) and tested them after shipment to the Netherlands.

We tested 56 human, 190 camel, 3 bat, 14 donkey, 15 cow, 15 sheep, and 15 goat serum samples for antibodies against MERS-CoV spike S1 using the protein microarray technique (*4*). We performed a virus neutralization test and a spike S1 protein–based ELISA (human serum samples only) to confirm the detection of MERS-CoV antibodies by protein microarray (*5*). In confirmatory tests, we included equal numbers of negative serum samples of the same species, when available. We considered samples positive if results of all tests were positive (protein microarray cutoff 1:20, 50% plaque-reduction neutralization titer cutoff 1:20, ELISA cutoff optical density 0.5). To resolve problems with possible mislabeling, we tested all animal serum samples collected in 2017 with a cytochrome B gene PCR to confirm species origins (*6*)*.* We tested camel nasal (n = 168), nasopharyngeal (n = 24), and rectal (n = 61) swab specimens and milk (n = 33), urine (n = 30), and fecal (n = 42) samples for MERS-CoV RNA using a reverse transcription PCR targeting the upstream of envelope and nucleocapsid genes, as described previously (*7*,*8*)*.* In addition, we tested legs of camel ticks (*Hyalomma dromedarii*) and bat (*Tadarida* spp.) tissues collected at the TCRC in 2015 for MERS-CoV RNA.

In 2015, a total of 92% of camels in Sudan and 99% of camels exported to Qatar from Sudan were MERS-CoV seropositive (Table). In 2017, all camels tested in Sudan were seropositive. No MERS-CoV antibodies were found in human or bat serum samples or serum samples from livestock other than camels. MERS-CoV RNA was detected in the nasal swabs from 3 camels imported into Qatar in 2015 but in no other samples.

**Table:** MERS-CoV RNA and antibody positivity among humans, bats, camels, and other livestock, Sudan, 2015 and 2017*

Sample type	Location of sample collection	2015				2017		
		No. samples	MERS-CoV RNA, no. positive/total no. (%)	MERS-CoV antibody, no. positive/total no. (%)		No. samples	MERS-CoV RNA, no. positive/total no. (%)	MERS-CoV antibody, no. positive/total no. (%)
Camel worker serum	Tamboul slaughter area	3	NT	ND		35	NT	ND
	Tamboul market	8	NT	ND		NA		
	TCRC	7	NT	ND		3	NT	ND
Camel serum†	Tamboul slaughter area‡§	4	ND	4/4 (100)		13	ND	13/13 (100)
	Tamboul market‡§¶	27	ND	26/27 (96)		NA		
	TCRC¶	31	ND	27/31 (87)		25	ND	25/25 (100)
	Qatar§¶#	90	ND	89/90 (99)		NA		
Camel nasopharyngeal swabs†	TCRC¶	NA				24	ND	NT
Camel nasal swabs†	Tamboul slaughter area‡§	11	ND	NT		NA		
	Tamboul market‡§¶	11	ND	NT		NA		
	TCRC¶	31	ND	NT		25	ND	NT
	Qatar§¶#**	90	3/90 (3)	NT		NA		
Camel ticks (*Hyalomma dromedarii*) from 25 camels	TCRC	155	ND	NT		NA		
Other animal serum								
Cattle	Tamboul slaughter area	6	ND	ND		9	NT	ND
Goat	Tamboul slaughter area	5	ND	ND		10	NT	ND
Sheep	Tamboul slaughter area	5	ND	ND		10	NT	ND
Donkey	Tamboul slaughter area	5	ND	ND		9	NT	ND
Bat††	TCRC	3	ND	NT		NA		
Bat tissue‡‡	TCRC	13	ND	NT		NA		

*MERS-CoV, Middle East respiratory syndrome coronavirus; NA, not available; ND, not detected; NT, not tested; TCRC, Tamboul Camel Research Centre. †From camels >2 years of age. ‡Serum and swab samples received from the slaughter field and live animal market were not matched. §Sample set included meat camels. ¶Sample set included milk camels. #Sample set included race camels. **Just imported into Qatar from Sudan. ††Genus unknown. ‡‡*Tadarida* spp.; lung, intestine, and brain tissues stored in formalin.

The results of this study are in agreement with other seroepidemiologic studies performed in Africa. The camel population was highly seropositive for MERS-CoV, and none or a low percentage of nasal or nasopharyngeal swabs from camels were positive for MERS-CoV RNA. As shown before in other countries in Africa, human serum samples did not show neutralizing activity against MERS-CoV (*2*). In 1 study in Kenya, 2 of 1,122 livestock handlers were found positive for MERS-CoV neutralizing antibodies (*9*). Other livestock were also seronegative for MERS-CoV in our study, a finding in agreement with most serosurveys, although some sheep, goats, and donkeys and 1 cow have been reported to have MERS-CoV antibodies (*3*,*10*).

The number of human and livestock samples tested was low in this investigation. Therefore, the results of this study are not conclusive. However, this study provides preliminary insight into MERS-CoV circulation in Sudan, the country with the third largest dromedary camel population in the world (http://www.fao.org/faostat/en/#data/QA). We show evidence of extensive MERS-CoV circulation in camels but no evidence of circulation in other livestock, bats, and humans.

## References

[1] Reusken CB, Raj VS, Koopmans MP, Haagmans BL. Cross host transmission in the emergence of MERS coronavirus. Curr Opin Virol. 2016;16:55–62. 10.1016/j.coviro.2016.01.004 26826951 PMC7102731

[2] So RT, Perera RA, Oladipo JO, Chu DK, Kuranga SA, Chan KH, et al. Lack of serological evidence of Middle East respiratory syndrome coronavirus infection in virus exposed camel abattoir workers in Nigeria, 2016. Euro Surveill. 2018;23. 10.2807/1560-7917.ES.2018.23.32.1800175 30107872 PMC6092911

[3] Sikkema RS, Farag EABA, Islam M, Atta M, Reusken CBEM, Al-Hajri MM, et al. Global status of Middle East respiratory syndrome coronavirus in dromedary camels: a systematic review. Epidemiol Infect. 2019;147:e84. 10.1017/S095026881800345X 30869000 PMC6518605

[4] Reusken CBEM, Haagmans BL, Müller MA, Gutierrez C, Godeke G-J, Meyer B, et al. Middle East respiratory syndrome coronavirus neutralising serum antibodies in dromedary camels: a comparative serological study. Lancet Infect Dis. 2013;13:859–66. 10.1016/S1473-3099(13)70164-6 23933067 PMC7106530

[5] Okba NMA, Raj VS, Widjaja I, GeurtsvanKessel CH, de Bruin E, Chandler FD, et al. Sensitive and specific detection of low-level antibody responses in mild Middle East respiratory. Emerg Infect Dis. 2019;25:1868–77. 10.3201/eid2510.190051 31423970 PMC6759241

[6] Kocher TD, Thomas WK, Meyer A, Edwards SV, Pääbo S, Villablanca FX, et al. Dynamics of mitochondrial DNA evolution in animals: amplification and sequencing with conserved primers. Proc Natl Acad Sci U S A. 1989;86:6196–200. 10.1073/pnas.86.16.6196 2762322 PMC297804

[7] Corman VM, Eckerle I, Bleicker T, Zaki A, Landt O, Eschbach-Bludau M, et al. Detection of a novel human coronavirus by real-time reverse-transcription polymerase chain reaction. Euro Surveill. 2012;17:20285. 10.2807/ese.17.39.20285-en 23041020

[8] Corman VM, Müller MA, Costabel U, Timm J, Binger T, Meyer B, et al. Assays for laboratory confirmation of novel human coronavirus (hCoV-EMC) infections. Euro Surveill. 2012;17:20334. 10.2807/ese.17.49.20334-en 23231891

[9] Liljander A, Meyer B, Jores J, Müller MA, Lattwein E, Njeru I, et al. MERS-CoV antibodies in humans, Africa, 2013–2014. Emerg Infect Dis. 2016;22:1086–9. 10.3201/eid2206.160064 27071076 PMC4880087

[10] Kandeil A, Gomaa M, Shehata M, El-Taweel A, Kayed AE, Abiadh A, et al. Middle East respiratory syndrome coronavirus infection in non-camelid domestic mammals. Emerg Microbes Infect. 2019;8:103–8. 10.1080/22221751.2018.1560235 30866764 PMC6455111

